# Integration of physical and mental health services for children and young people with eating disorders and functional symptom disorders: discrete choice experiment

**DOI:** 10.1186/s12913-024-12157-8

**Published:** 2025-01-03

**Authors:** Efthalia Massou, Mike Basher, Sophie D. Bennett, Tamsin Ford, Saheli Gandhi, Isobel Heyman, Josefine Magnusson, Raj Mehta, Pei Li Ng, Sara O’Curry, Angus I. G. Ramsay, Naomi J. Fulop, Stephen Morris

**Affiliations:** 1 Primary Care Unit, Department of Public Health and Primary Care, University of Cambridge, Cambridge, CB1 8RN UK; 2 Cambridgeshire and Peterborough NHS Foundation Trust, Elizabeth House, Fulbourn, Cambridge, CB21 5EF UK; 3 UCL Great Ormond Street Institute of Child Health, 30 Guilford Street, London, WC1N 1EH UK; 4 Institute of Psychiatry, Psychology & Neuroscience, King’s College London, De’Crespigny Park, London, SE5 8AF UK; 5 Department of Psychiatry, Hershel Smith Building Cambridge Biomedical Campus, Cambridge, CB2 0SZ UK; 6 Department of Applied Health Research, University College London, Gower Street, London, WC1E 6BT UK; 7 Cambridge Children’s Hospital Project Team, Cambridge University Hospitals NHS Foundation Trust, Hills Road, Cambridge, CB2 0QQ UK; 8 Paediatric Psychological Medicine, Cambridge University Hospitals NHS Foundation Trust, Hills Road, Cambridge, CB2 0QQ UK; 9 Department of Women and Children’s Health, King’s College London, St Thomas’ Hospital, Westminster Bridge Road, London, SE1 7EH UK; 10 Patient representative, London, UK

**Keywords:** Integration, Eating disorders, Functional symptom disorders, Discrete choice experiment, Preferences

## Abstract

**Background:**

Given the increasing recognition of the value of greater integration of physical and mental health services for children and young people, we aimed to evaluate preferences among parents for the characteristics associated with integrated health service provision for two conditions (eating disorders, functional symptom disorders).

**Methods:**

Two discrete choice experiments (DCEs) were conducted, using electronic surveys. Participants were adult parents of children and young people. Choice scenarios were based on five attributes for the eating disorders study, and four attributes for the functional symptom disorders study.

**Results:**

Two hundred parents participated in each DCE. For eating disorders, days missed from school in the last year was the attribute valued most highly, followed by days in hospital in the last year, costs to the NHS, functioning, and interaction with peers with eating disorders. Respondents were willing to trade £531 of costs to the NHS for one less day missed from school. For functional symptom disorders, time to diagnosis was valued most highly, followed by days missed from school while obtaining a diagnosis, reservations about seeing a mental health practitioner, and costs of diagnosis to the NHS. Respondents were willing to trade £4237 of costs to the NHS to wait one month less for a diagnosis.

**Conclusion:**

Respondents’ preferences were largely consistent with the planned goals of integrating physical and mental health services. Our findings show the factors which ought to be considered when designing new integrated pathways and evaluating them.

**Supplementary Information:**

The online version contains supplementary material available at 10.1186/s12913-024-12157-8.

## Background

Traditionally, children and young people requiring specialist health care were cared for by a single specialist, who could address the majority of their immediate health care needs, with mental and physical health care delivered by separate services as required [1]. More recent trends in care involve greater collaboration between specialists, with more multidisciplinary care for children and adolescents with long-term health conditions and disabilities [1]. This trend is a response to an increasing recognition of the importance of holistic care for the child, that addresses both physical and mental health care needs, to facilitate adjustment and coping, increase adherence and thus optimise treatment response. This approach addresses distress and mental health issues early, and helps the child or young person live as full and healthy a life as possible. There is growing awareness of the extent of mental health difficulties such as anxiety and depression in young people in general, and that these are particularly common among those with complex chronic health conditions and disabilities [2]. Conversely, there is increasing evidence that children and young people with mental health problems are at risk of physical health conditions, such as infectious diseases, respiratory problems, and weight-related problems [3].

A recent review described three levels of collaboration between physical and mental health services [1]. The first level of co-ordination, with minimal collaboration, involves efforts to promote communication between individual medical and psychiatric health care providers. The second level involves the co-location of services. The third level is integrated care, where physical and mental health services co-ordinate care, including shared medical records and multidisciplinary care. There are different ways in which physical and mental health services might be integrated. Examples include specialist paediatric eating disorder services, where multidisciplinary treatment is increasingly the norm, or having psychologists based within epilepsy clinics, so that adjustment, coping or adherence problems can be picked up and addressed early, before they develop a secondary, negative impact on a young person’s quality of life, mental health or ability to participate in school or social activities [4, 5].

Depending on symptoms and illness presentation, children and young people may be seen by either physical or mental health teams when first assessed and treated. Eating disorders encompass a range of disorders related to eating and feeding [6]. Treatment is usually led by mental health services, with contact with physical health services typically limited to weight monitoring, blood tests and responding to worsening, and sometimes life-threatening, physical difficulties arising as a consequence of the disorder. Functional symptom disorders (sometimes referred to persistent physical symptoms disorders or medically unexplained symptoms) is the name given to physical symptoms for which there is no obvious physical cause [7]. These disorders are often assessed first by physical health services but then later passed on to mental health services after multiple tests have ruled out serious underlying disease.

The current study was conducted as part of an independent evaluation of the ongoing development of a new children’s hospital in England. A key factor behind the re-development of services associated with the new hospital is greater integration between physical and mental health services, to be facilitated by the co-location of services. To support this development, it is helpful to understand the factors that matter to people when designing new integrated care pathways and assessing them, for example to improve staff and user experience and uptake [8]. The aim of this study was to examine parental preferences for integrated care. The intention is to aid in the development of this care by designing pathways that specifically aim to improve the factors that are important to parents and give us a means of assessing whether the pathways achieve their stated aims. We focused on the integration of physical and mental health services for eating disorders and functional symptom disorders. These are conditions typically requiring input from both physical and mental health services, but in different ways, as described above. To our knowledge there are no previous studies that have examined this topic.

## Methods

### Overview of approach

Preferences for integrated pathways for the two conditions were explored with two discrete choice experiments (DCEs) using online surveys [9]. In DCEs, respondents are typically presented with a series of questions, asking them to choose between two or more alternatives that describe an option (e.g., a service or technology or pathway) in terms of a set of characteristics or attributes. This allows the attributes of the option that respondents prefer to be evaluated, as well as the trade-off they are willing to make between these attributes. DCE good-practice guidelines were followed for the design and analysis of this study [10].

### User involvement

The study was supported by a Project Advisory Group (PAG), comprising five health care professionals involved in the organisation and delivery of care for children and young people with eating disorders and functional symptom disorders and with an interest in integrating physical and mental health services. It was also supported by a Patient and Public Involvement Advisory Group (PPIAG), comprising four parents of children and young people affected by either an eating disorder or functional symptom disorder. The Study Protocol is available at https://fundingawards.nihr.ac.uk/award/NIHR133613.

### Attributes and levels

The attributes and levels used in the DCEs were identified using three sources. First, a systematic review of integrated mental and physical health services for children and young people with eating disorders and functional symptom disorders [11]. Second, with input from the study PAG and PPIAG. Third, we conducted two virtual interviews with health care professionals, one involved in the organisation and delivery of care for children and young people with eating disorders, the second with functional symptom disorders.

Analyses of the data from these sources identified five attributes that might matter to stakeholders when considering preferences for the integration of services for eating disorders. These attributes were chosen to reflect key aspects of care pathways that are either supported by integration or might be impacted by its absence. The five attributes were: days missed from school in the last year; days in hospital in the last year; functioning; costs to the NHS; and interaction with peers with eating disorders. Days missed from school capture the impact of care on a child’s or young person’s education, with integrated care aiming to minimize disruptions by addressing effectively both the physical and mental health challenges and ensuring child’s regular attendance to school. Days in hospital highlight the effectiveness of care. A high number of days in hospital may suggest that physical and mental health issues are not being managed cohesively, leading to repeated admissions or prolonged stays. Integrated care pathways aim to reduce hospitalisation by proactively managing the physical and mental health challenges based on coordinated community-based and outpatient services. Functioning used as a measure of child’s overall health and ability to engage in normal activities and maintain independent. Functional limitations often arise from both physical complications (e.g., malnutrition, fatigue) and mental health challenges (e.g., anxiety, depression). Improvements in functioning are likely to result from pathways that holistically address these interconnected aspects through integrated care approaches. The attribute regarding the costs to the NHS represents the financial efficiency of the care pathway. Lower costs might indicate streamlined, integrated services that avoid duplication or inefficiencies and optimize resource use, whereas higher costs could reflect fragmented or poorly coordinated care requiring redundant assessments or interventions. Evidence regarding expectations from integrated care in these attributes is derived from a previously published studies [12, 13] and was in line with the input from the study PAG and PPIAG.

We identified four attributes in the context of preferences for the management of functional symptom disorders: reservations about seeing a mental health practitioner; time to diagnosis; costs of diagnosis to the NHS; and days missed from school while obtaining a diagnosis (Table 1). The first attribute represents parental concerns or hesitations regarding their child accessing mental health services, such as stigma or doubts about the legitimacy of psychological explanations for physical symptoms. High levels of reservation may indicate barriers to integration, where mental health care is perceived as separate from or optional within overall health services. This binary approach to healthcare—categorizing children and young people as either needing mental health support or not—can perpetuate stigma. Conversely, pathways designed to reduce such reservations, through normalization and coordination of mental and physical health services, align with the goals of integrated care. The time to diagnosis is a measure of the efficiency and coordination of care pathways. Shorter times to diagnosis are likely achieved when physical and mental health teams work closely together to streamline assessments and avoid unnecessary delays. Longer times might suggest fragmented care, where patients navigate separate systems without cohesive support [14]. The rationale behind the last two attributes is the same with that described in the case of eating disorders.

**Table 1:** Attributes and levels used in the discrete choice experiments

**Attribute**	**Description**	**Levels**				
**Eating disorders DCE**						
Days missed from school in the last year	Refers to how the child's/young person’s eating disorder and the care they receive for it disrupts their education due to days missed from school.	10 days	25 days	50 days	100 days	
Days in hospital in the last year	Refers to the time spent in hospital or other facility by the child/young person due to their eating disorder and the care they receive for it.	10 days	50 days	100 days		
Functioning	Describes how the child/young person is able to function in everyday life as a consequence of their eating disorder and the care they receive for it. For example, everyday functioning may be affected due to the physical and mental health repercussions of eating disorders (e.g., stomach cramps, dizziness, fainting spells, muscle weakness, anxiety and depression).	There is some impact on everyday functioning; some usual activities are not undertaken or require assistance.		There is severe impact on everyday functioning; usual activities are not possible, and the child/young person is unable to be independent.		
Costs to the NHS	Describes how much the NHS have to pay per year for the care provided to the child/young person for their eating disorder.	£1000	£10000	£25000	£40000	
Interaction with peers with eating disorders	Describes the impact that the care the child/young person receives has on interacting with peers with eating disorders. This has potential negative effects, for example, by increasing exposure to negative views about body image, and competition between peers to lose weight.	There is minor or occasional interaction with peers with eating disorders.		There is frequent or constant interaction with peers with eating disorders.		
**Functional symptom disorders DCE**						
Reservations about seeing a mental health practitioner	Refers to extent of reservations about the child/young person seeing a mental health practitioner (e.g., a psychologist or psychiatrist) and the possibility of them requiring psychological/ psychiatric treatment. These reservations might arise from the fear of stigma and prejudice regarding mental health conditions, and the concerns that the symptoms the child/young person experience are not “real”.	I have some reservations about the child/young person seeing a mental health practitioner.		I have major reservations about the child/young person seeing a mental health practitioner.		
Time to diagnosis	Refers to the length of time needed from initial contact about the child’s/young person’s symptoms with primary care until a final diagnosis is obtained. This does not include potential diagnoses that are explored across the diagnostic pathway.	3 months	6 months	12 months	18 months	24 months
Costs of diagnosis to the NHS	Refers to the costs borne by the NHS to reach a diagnosis for the child/young person, for example arising from the number of clinic visits and diagnostic tests needed to reach a diagnosis	£1000	£5000	£10000	£15000	
Days missed from school while obtaining a diagnosis	Refers to how the child's/young person’s symptoms and the health care contacts they receive for these disrupts their education due to days missed from school. This covers the time from the child/young person first experiencing their symptoms until a final diagnosis is obtained	10 days	20 days	30 days	50 days	

Levels for each attribute were chosen using a combination of approaches to ensure their relevance and appropriateness. Where available, we referred to published data on related aspects, such as the average days of school missed by children and young people which was used to estimate the absence from school. In this case, we considered that students in the UK attend 190 school days per year. Persistent absence was estimated at around 7 days per term, while severe absence was approximately 95 days (equivalent to 50% of school sessions). For the DCE questionnaire on eating disorders, this provided a range of 10–100 days, divided into three categories. In the case of functional symptoms, this range was adjusted to 10–50 days since the attribute was referred to the days missed from school until the final diagnosis, excluding the treatment. For functional symptoms, this range was adjusted to 10–50 days, as the attribute referred to days missed from school until the final diagnosis, excluding treatment. To capture variations in school absenteeism within this shorter range, we considered four levels (10, 20, 30, and 50 days). This approach was consistent with input from the study PAG and PPIAG.

Regarding days in hospital due to eating disorders, substantial variability was observed in reported values, influenced by country, demographic characteristics, and year of publication. The range of 10–100 days used in our study was derived from literature that closely aligned with our target population and was thoroughly discussed with the study PAG and PPIAG. For instance, Morris et al. [15] reported a mean length of stay of 141 days in a sample of adolescents in Scotland, while Kan et al., in a meta-analysis [16], calculated a mean of 76 days. Evidence from the latter study, focusing on children and young people, showed values close to our range, with only a few extreme cases exceeding 180 days [17].

For the typical costs to the NHS associated with care for eating disorders we took into consideration reported costs per person per year ranging from approximately £1000 to £42,000, when psychiatric services were required [18]. For the typical costs to the NHS associated with functional symptoms, we relied on existing resource use estimates which, however, were based on adult populations [19]. Recognizing the limited availability of specific data in some areas as well as the amount of assumptions and approximations that we need to do, we relied meaningfully on input from the study PAG and PPIAG and stakeholder to guide level selection. This iterative and consultative process ensured that the levels reflected plausible and meaningful variations in care pathways, even where direct literature evidence was not available. Descriptions were developed for each attribute to help participants understand the nature of each attribute that they were being asked to consider. All material was scrutinised by the PAG and the PPIAG, who made changes to the descriptions of the attributes, and the levels.

### Questionnaire design

**Fig. 1:**
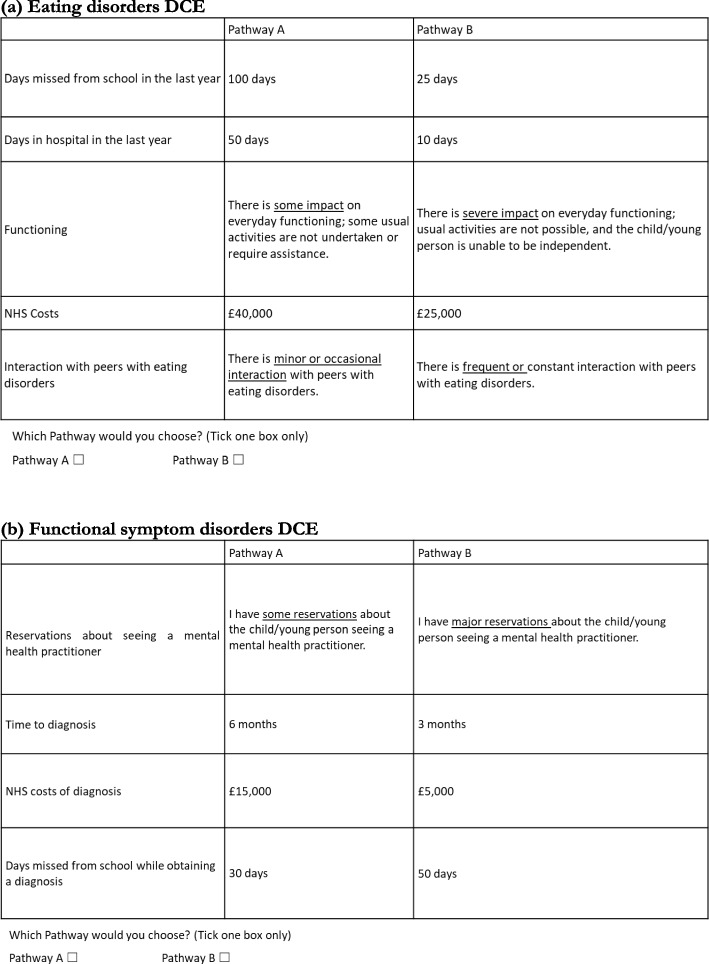
Examples of DCE questions

For both conditions, respondents were asked to choose their preferred option from a series of pairwise choices, asking in which of two fictitious pathways they would prefer their child to receive their care. Each service was described by a combination of different levels of the attributes; Fig. 1 shows example of DCE questions for both conditions. An opt-out or ‘neither’ option was not included. For the eating disorders DCE the number of potential combinations of attributes with two four-level attributes, one three-level attribute and two two-level attribute was 192 (4 × 4 × 3 × 2 × 2). With two options to choose from in each choice question, this gives a possible 36,672 choices (192 × 191). For the functional symptom disorders DCE the number of potential combinations was 160 (5 × 4 × 4 × 2), giving a possible 25,440 choices (160 × 159). To reduce the number of choices in both DCEs to a manageable number, a fractional design was applied using the –dcreate– command in Stata SE v18.0, which creates optimally efficient designs for DCEs within the constraints imposed [20]. In both DCEs the choice set was reduced to 16 scenarios, which were split into two blocks of eight, and half of the respondents in each DCE were assigned to each block. The questionnaire also included a question asking respondents to rank the attributes according to their overall importance, from most to least important, as well as information on demographic and socioeconomic factors. The questionnaire was piloted by our PPIAG and PAG plus two health care professionals, who provided verbal and written feedback, which improved the wording and appearance of the questionnaire. The final version of the questionnaire that we developed for each condition and each block, is given in the Supplementary Material, Appendices I-IV.

### Survey sampling

Participants in the two DCEs were adult parents and carers of children and young people aged 0–18 years. They did not necessarily have direct experience of eating disorders or functional symptom disorders. We aimed to recruit 200 participants for each DCE, 400 in total. Although no consensus exists regarding sample size calculation for DCEs because of their complexity (such as number of attributes and levels), we intended the sample size to be similar to previous studies [21, 22]. An independent survey company (IQVIA) was hired to administer the online questionnaire to a representative sample of the general parent and carer population of the UK. The company created an electronic version of the survey using a bespoke online platform. Potential participants were recruited by a third-party panel provider, Dynata. They were sent a weblink to the survey by email that had an embedded Participant Information Sheet at the start. From this, participants were asked to click to another webpage to access the survey and were informed that by doing so they consented to take part in the study. They were also told that they did not have to take part if they did not want to. There were no restrictions on participants in terms of demographic factors (other than being a parent or carer of a child or young person aged 0–18 years), or geographical location within the UK. Panel members were highly unlikely to have completed both DCEs. All participants gave informed consent prior to participating in the study and received a small incentive in the form of panel points (equivalent of £2.90) for completion of the questionnaire. The questionnaire was online for six days in August 2023, when the target numbers of 200 respondents for each DCE was reached.

### Data analysis

Descriptive statistics for the characteristics of respondents in each DCE were computed. Responses to the ranking questions were analysed graphically. The DCE data were analysed using mixed logit regression models in which the outcome was pathway preference (A or B) and the variables in the equation were the individual attributes. We first ran models assuming the coefficients on all of the attributes were fixed. To assess preference heterogeneity among participants we then ran models specifying the coefficient on each attribute in turn to be random, with all the others fixed. We ran likelihood ratio tests for each of these models, testing the null hypothesis that all the coefficients were fixed. After testing all attributes individually for preference heterogeneity in this way, we then ran a final model. In that model, the coefficients on all the attributes where the previously tested null hypothesis (i.e., that the coefficients were fixed) was rejected, were allowed to be random. In these final models we allowed the multiple random coefficients to be correlated, assuming a multivariate normal distribution. The relative importance of each attribute was calculated as the difference in the coefficients between the best or most preferred level of each attribute and the worst or least preferred level of the same attribute [23].

We calculated marginal rates of substitution (MRS) with respect to the cost attribute (costs to the NHS, cost to the NHS of diagnosis) for attributes found to be significant predictors of preferences; this allows direct assessment of how much of one attribute participants are willing to trade for one unit of another attribute, and therefore enables a comparison of different attributes on a common scale, in this case costs to the NHS. This approach was chosen because the context of our study involves evaluating preferences for publicly funded services within the NHS, where families would not pay for the services themselves. Thus, it captures the societal or system-level resource valuation, in other words the perceived valuation of the services and how resources should be prioritized within the healthcare system, rather than direct consumer spending. It means that the MRS does not reflect actual consumer willingness to pay. This is an approach that has been used elsewhere [24]. All analyses were undertaken using the software package Stata® SE version 18.0 (StataCorp, College Station, Texas, USA).

## Results

### Responses and sample

Four hundred responses to the DCEs were received, 200 for each DCE. In both DCEs the modal age group for respondents was 35–44 years (around 40% of both samples; Table 2). In the eating disorders DCE there were slightly more males than females (49% versus 46%) and the opposite was true for the functional symptom disorders DCE (42% versus 53%). Around two-thirds of both samples were single carers, and 80% + were in the white ethnic group. The modal education category was degree or higher degree, or equivalent (around 45% of both samples). There was representation from across all regions of the UK in both groups.

**Table 2:** Sample characteristics

	**Eating disorders DCE (*N*** **= 200)**		**Functional symptom disorders DCE (*N*** **= 200)**	
	**n**	**%**	**n**	**%**
**Age group**				
18–24 years	6	3	5	2
25–34 years	50	24	43	21
35–44 years	81	39	83	40
45–54 years	44	21	49	24
55 + years	21	10	20	9
Prefer not to say	0	0	0	0
Missing	7	3	7	3
**Sex**				
Female	97	46	110	53
Male	102	49	87	42
Other/Prefer not to say/Missing	10	4	10	5
**Single carer**				
Yes	128	61	117	57
No	71	34	79	38
Prefer not to say/Missing	10	5	11	5
**Ethnic group**				
White	180	86	165	80
Indian/Pakistani/Bangladeshi	15	7	11	5
Black, African, Black Caribbean, Black British	4	2	9	4
Other/Prefer not to say/Missing	10	4	22	10
**Education**				
No formal qualifications, or equivalent	4	2	2	1
O level or GCSE, or equivalent	34	16	39	19
ONC or BTEC, or equivalent	5	2	8	4
A level (‘Higher’ in Scotland), or equivalent	41	20	28	14
Higher-education qualification below degree, or equivalent	22	11	25	12
Degree or higher degree, or equivalent	94	45	97	47
Prefer not to say/Missing	9	4	8	4
**Region of residence**				
East of England	20	10	15	7
East Midlands	12	6	13	6
London	31	15	39	19
North East & Cumbria	11	5	12	6
North West of England	23	11	28	14
Scotland	18	9	19	9
South East of England	26	12	25	12
South West of England	11	5	5	2
Wales & Northern Ireland	10	5	8	4
West Midlands	17	8	12	6
Yorkshire	22	11	24	12
Prefer not to say	0	0	0	0
Other	0	0	0	0
Missing	8	4	8	4

Categories are combined where there are 1–3 observations

### Simple attribute ranking

**Fig. 2:**
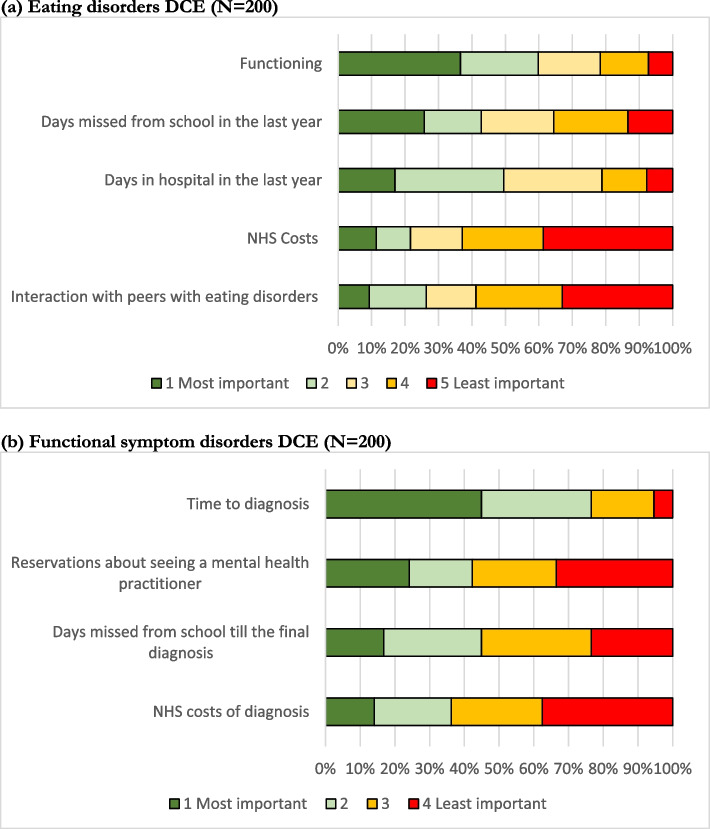
Ranking of attributes

The responses to the ranking question posed after the DCE questions in both questionnaires were examined (Fig. 2). Attributes were ranked by likelihood of being selected as the most important factor; using this method of ranking, functioning and days missed from school in the last year were ranked highly by respondents to the eating disorders DCE, and costs to the NHS and interaction with peers with eating disorders were ranked to be the least important factors. For the functional symptom disorders DCE time to diagnosis was ranked as the most important factor, and cost to the NHS of diagnosis as the least important factor.

### Regression analysis

In the eating disorders DCE we failed to reject the null hypothesis of fixed coefficients for all of the attributes. As expected, respondents preferred pathways resulting in fewer days missed from school, where costs to the NHS were lower, where there was less severe impact on functioning, and where there were fewer days in hospital (Table 3). Interaction with peers with eating disorders was not a significant factor affecting preferences in this sample. In the functional symptom disorders DCE, based on the fixed coefficients, respondents preferred pathways with a shorter time to diagnosis, with fewer days missed from school while obtaining a diagnosis, where there were fewer reservations about seeing a mental health practitioner, and where the costs to the NHS of diagnosis were lower. We rejected the null hypothesis of fixed coefficients for the time to diagnosis and days missed from school while obtaining a diagnosis attributes. The estimated mean of the normally distributed coefficients on time to diagnosis was − 0.201 and on days missed from school while obtaining a diagnosis it was −0.087. The estimated standard deviations of these random coefficients were 0.600 and 0.311, respectively, indicating heterogeneity across individuals in the sample with respect to the effect of both attributes.

**Table 3:** Results of mixed logit regression analyses

	**Coefficient**	**(95% CI)**	**LR test**	**Mean of random coefficients**	**SD of random coefficients**	**MRS**	**RI**
**Eating disorders DCE (*N* = 202)**							
Days missed from school in the last year	−0.007	(−0.010, −0.005)	0.49			−531	(1)
Costs to the NHS	−0.00001	(−0.00002, −0.00001)	0.50				(3)
Functioning							(4)
Some impact	Base						
Severe impact	−0.524	(−0.629, −0.419)	0.48			−37,166	
Days in hospital in the last year	−0.007	(−0.010, −0.003)	0.34			−480	(2)
Interaction with peers with eating disorders							(5)
Minor or occasional interaction	Base						
Frequent or constant interaction	0.028^a^	(−0.077, 0.133)	0.48			-	
Number of observations	3232						
**Function symptom disorders DCE (*N* = 200)**							
Time to diagnosis	−0.026	(−0.036, −0.017)	< 0.01	−0.201	0.600	−4237	(1)
Days missed from school while obtaining a diagnosis	−0·010	(−0·015, −0·006)	< 0·01	−0·087	0·311	−1672	(2)
Reservations about seeing a mental health practitioner							(3)
Some reservations	Base						
Major reservations	−0.299	(−0.403, −0.195)	0.50			−48,048	
Costs of diagnosis to NHS	−0.000006	(−0.00007, −0.00005)	0.50				(4)
Number of observations	3200						
Correlation coefficient (95% CI)	−0.179	(−0.812, 0.648)					

The column ‘LR test’ shows the results of the likelihood ratio tests versus fixed coefficients for each attribute; values reported are *P*-values; values < 0·05 imply we can reject the null hypothesis that the coefficients on are fixed. We report the mean and standard deviation of the estimated coefficients for the random coefficients where we reject the null hypothesis that the coefficients are fixed. The random coefficients are allowed to be correlated, assuming a multivariate normal distribution; the correlation coefficient is reported in the bottom row. The numbers column ‘RI’ show the ranking of attributes in terms of their relative important with (1) being most important, (2) being next most important, etc.

See text for calculation of the MRS and RI. The coefficients are rounded and therefore the MRS values are not identical to the ratio of the coefficients shown in the table

*CI* confidence interval, *SD* standard deviation, *MRS* marginal rate of substitution (£), *RI* relative importance, *LR* likelihood ratio

^a^Coefficient not significantly different from 0; all other coefficients are significant at *P* < 0·01

### Relative importance of the attributes

Over the range of levels included in the eating disorders DCE, days missed from school in the last year was the attribute valued most highly by respondents, followed by days in hospital in the last year, then costs to the NHS, then functioning, followed by interaction with peers with eating disorders (Table 3). For the functional symptom disorders DCE, time to diagnosis was valued most highly, followed by days missed from school while obtaining a diagnosis, then reservations about seeing a mental health practitioner, followed by costs of diagnosis to NHS. These analyses of the relative importance of the attributes are preferred to the simple attribute ranking as they account for the levels of the attributes.

### Marginal rates of substitution

As an indication of their strength of preference, and the value they put on each attribute, respondents in the eating disorders DCE were willing to trade £531 of costs to the NHS for one less day missed from school; £37,166 of costs to the NHS to avoid severe impacts on functioning; and £480 of costs to the NHS for one less day in hospital (Table 3). Respondents in the functional symptom disorders DCE were willing to pay £4237 of costs to the NHS to wait one month less for a diagnosis; £1672 of costs to the NHS for one less day missed from school while waiting for a diagnosis, and £48,048 of costs to the NHS to avoid major reservations about seeing a mental health practitioner.

## Discussion

### Key findings

We carefully selected attributes and their levels for the two DCEs to capture different aspects of the care pathways for children and young people. Both DCEs were designed to reflect the potential characteristics of pathways, whether they represent integrated care—where physical and mental health services are closely coordinated—or more fragmented models of care.

By examining the levels of each attribute, the study aimed to identify the preferences for pathways that align with the principles of integration versus those that may indicate uncoordinated or standalone approaches to service provision. Participants were adult members of the general public with caring responsibilities for children and young people. In both DCEs, participants preferred pathways that resulted in fewer days absence from school and fewer days in hospital. Additionally, in the eating disorders DCE, participants preferred lower costs to the NHS, and lower impact on functioning and usual activities. Interaction with peers with eating disorders was found to not significantly affect preferences. In the functional symptom disorders DCE, participants preferred a shorter wait to receive a diagnosis and having fewer reservations about seeing a mental health practitioner. The latter was included in the DCE as an attribute of the service to capture how potential barriers related to service interaction might affect the decision to seek care. While it may seem related to personal attitudes, we framed it as an aspect of the service because it represents an external barrier that might cause hesitation in seeking help. This attribute addresses the service environment's ability to reduce perceived barriers or hesitations in accessing mental health care, making it an important functional component of the service offering. It aligns with prior studies that treat such barriers (e.g., stigma or discomfort related to accessing care) as modifiable aspects of the service provision rather than just individual respondent characteristics [25]. Moreover, these barriers can be influenced by the structure, communication, and approach of the mental health service itself, highlighting the functional aspect of this attribute within the service design. The attributes and levels used in both DCEs collectively offer insights into how different care models might address the multifaceted needs of children and young people with eating disorders or functional symptoms. Preferences for less severe impact on education, and everyday activities along with lower costs to the NHS, were found to be largely consistent with the goals of integrating physical and mental health services, which aims to improve outcomes and reduce both costs and patient and family burden.

### How the findings relate to previous research

There are several studies that have explored preferences for integrated care, though these have tended to focus on which outcomes matter most to people with multiple chronic disease/multi-morbidities [26, 27]. We are not aware of any DCE studies that have evaluated preferences for integration of physical and mental health services for children and young people with these two conditions.

### Limitations

DCEs elicit hypothetical choices, and therefore might lack external validity if individuals do not make the same choices in real-life situations. Some aspects of the DCE might be difficult for respondents to understand, such as the forced choices between pathways, probabilities and clinical concepts. A potential limitation of this study is the presence of social desirability bias which might compromise the validity of the findings. Our samples were members of the general public with children, though not necessarily of children affected by eating disorders or functional symptom disorders. However, all children and young people are at risk of becoming affected by these conditions, meaning the preferences of the respondents are valid and informative in terms of planning pathways for integrated care in children and young people affected by these conditions. The probable lack of lived experience may explain why, for example, interaction with peers with eating disorders was found to not significantly affect preferences among the eating disorders DCE, given that peer influence is found to have a negative influence on eating disorder outcomes [28]. Additionally, the representativeness of the samples used might be limited by the recruitment strategies, yielding potential sampling bias. For example, there was a high proportion of single carers in our sample. Additionally, there were no screening questions for participating in the DCE and the response rate is not known given that we used an online panel. The modal education category was those who were educated to degree level or higher, and it is unclear if costs would have been the least important attribute if, for example, the sample was on average less well educated. There might be other components of integrated care that are important but were not included in the present analysis. The levels of the attributes were identified considering options in between the minimum and the maximum values that we found in literature. This consideration lacks evidence regarding the validity of the levels. Unfortunately, the number of attributes and that of levels that can be included in a DCE is limited by the amount of data that participants can process. Avoiding increased complexity from adding more attributes and levels may limit the effectiveness of extrapolating results. The nature of our piloting work meant we were unable to produce initial estimates of the model coefficients, which could have been used to inform the final study design. Initially, the coefficient parameters were assumed to be zero. Preferences might vary by sub-groups within our samples of respondents (e.g. by education or ethnic group), but sample size considerations make sub-group analyses problematic. In addition, we acknowledge two limitations regarding the marginal rates of substitution. First, the marginal rates of substitution were, by definition, reflective of the chosen levels for the attributes. Second, the fact that in the functional symptom disorders DCE the marginal rate of substitution to avoid major reservations about seeing a mental health practitioner exceeds the levels of costs to the NHS indicates that the participants are willing to make trade-offs beyond what was anticipated by the design of the experiment. This might be due to design constraints, such as not capturing the full range of preferences for costs to the NHS, and might affect the practical applicability of the result.

### Implications of the findings

Recent studies have demonstrated that numbers of children being admitted to hospital with combinations of physical and mental health problems have increased. This highlights the need to design integrated care pathways as these complex patients require increased length of stay and greater resource [29]. Information about the preferences identified in this study could be used in the development of new integrated care pathways and services aimed at specifically improving the outcomes shown to be of importance to potential service users. For example, in the case of eating disorders, our findings suggest that there could be a focus on designing integrated pathways to reduce time off from school, costs to the NHS, and days in hospital, and to improve everyday functioning and ability to undertake usual activities. In the case of functional symptom disorders, the findings suggest that there could be a focus on designing integrated pathways to reduce time to diagnosis, days off school while waiting for a diagnosis, as well as costs to the NHS of obtaining a diagnosis. Reservations about seeing a mental health practitioner were also an important factor affecting preferences, suggesting that providers should seek to better understand and ameliorate these concerns among parents. The preferences identified in this study can also be used in health service research into integrated care pathways; this study has identified outcomes that matter to parents, which should be measured in future evaluations.

### Further research

This study provides new evidence on the elements of integrating physical and mental health services for children and young people affected by eating disorders and functional symptom disorders that matter to parents. Further research would be beneficial to evaluate integrated pathways, focussing in particular on the impact of those pathways on the attributes found to matter most to participants in this study. Further research to explore if the preferences among the general public sample used in the present study were consistent among patient and families with lived experience of the two conditions would also be beneficial.

## Conclusion

Respondents’ preferences in these two DCE samples were consistent with the goals of integrating physical and mental health services. This does not arise from a specific attribute but rather from the level of the attributes that reflect the extent of the integration. The findings are relevant to those who are designing integrated care pathways for children and young people affected by the two conditions, showing the factors which ought to be considered when designing new pathways and when evaluating them.

## Supplementary Information

Supplementary Material 1.

## Data Availability

The survey company and UCL signed a Data Sharing Agreement before data collection and the survey company appropriately sent to UCL these data. All data requests should be submitted to the corresponding author for consideration. Access to anonymised data may be granted following review.

## Abbreviations

DCE: Discrete Choice Experiment
NIHR: National Institute for Health and Care Research
PAG: Project Advisory Group
PPIAG: Patient and Public Involvement Advisory Group
NHS: National Health Service
