# Supplementary material for: Integration of physical and mental health services for children and young people with eating disorders and functional symptom disorders: discrete choice experiment
Source: BMC Health Serv Res. 2025 Jan 3;25:11. doi: 10.1186/s12913-024-12157-8 (PMC11697723; doi:10.1186/s12913-024-12157-8)
Supplement: Supplementary file 1 — Supplementary Material 1. [file 12913_2024_12157_MOESM1_ESM.docx]

**Supplementary Material**

**Appendix I. Questionnaire for eating disorders – Block 1**

**Title of Study:** Integration of specialised services for eating disorders and functional symptoms in children and young people (CENT): a mixed methods study

**Why do we need your help?**

We are interested in informing the planned integration of physical and mental health services for children and young people with eating disorders at Cambridge Children Hospital. The information you provide will be vital in helping us understand which issues matter to you the most, and how strongly you feel about these. Based on the findings we will inform the organisation of specialist services for this condition at Cambridge and elsewhere in the NHS.

**What we would like you to do?**

In the following pages we have listed some descriptions of imaginary pathways (i.e., hypothetical care pathways), which children and young people with an eating disorder might follow. These pathways are presented in pairs, and we would like you to think about these and tell us which one sounds best to you. There is no right or wrong answer. It is possible that in some of the pairs neither of the two options exactly matches your preferences, but please don’t worry about this. Your responses should reflect your views as a parent or carer of a child or young person (regardless of any health conditions). We know some of the choices might be hard, and you might sometimes have to choose the least bad option rather than choosing one which you would actually like. Please just do your best.

# Completing the survey

Please follow the instructions and complete all of the questions in each section using the spaces provided. If you make a mistake, simply change your selection to the correct answer. You can return to a previous question using the back arrow if you want to do so.

**Taking part in this survey is voluntary. Please note you must be aged 18 years or over to take part. The research team will not be able to link your responses back to you in any way. IQVIA is the company administering the survey on behalf of the research team. Please do not put your name or address anywhere on the questionnaire.**

Finally, thank you for participating in the survey, we really do appreciate the time you give up in order to share your experiences with us and we sincerely value all the information provided.

THE CENT RESEARCH TEAM

**IMPORTANT INFORMATION**

**By completing this questionnaire, you are giving your consent for the following:**

- You are providing data to IQVIA via this questionnaire. IQVIA will not use your data for any other purpose than this specific study and will not analyse your data.
- You will not be asked to provide any information that identifies you whilst completing the questionnaire online. Once you have submitted your answers, all the responses will be anonymised by IQVIA. After this you will not be able to ask for your data to be removed because it will not be possible to work out which data are yours.
- Fully anonymous completed questionnaires will then be sent by IQVIA to University College London, UK, where they will be analysed by researchers who are part of the research team.
- Nothing will be linked to any other health care information about you that is held by any health care provider, at any point.

Your answers to the questionnaire will be processed for the purposes outlined in this notice. By clicking on the consent to participate button below you are agreeing that you have read and understood the information contained in the participant information sheet.

START THE SURVEY

# Section A - Suitability for survey

Please read the following statements carefully and tick the appropriate box.

|  | YES | NO |
| --- | --- | --- |
| I am aged 18 years or over. |  |  |
| I am parent or carer of a child or young person |  |  |
| I have read and understood the information regarding the survey. |  |  |
| I understand that by completing this questionnaire, I am giving consent for any personal details that might have been used for contact purposes, to be held by a survey company before final anonymised dataset will be sent to UCL for the purposes of this study as outlined in the information provided. |  |  |
| I am aware of who I should contact if I wish to discuss any aspect of the study. |  |  |

If you answered NO for any of the statements, you are unfortunately not eligible to take part in the survey.

# Section B – Discrete Choice Survey

In the following section you will see eight pairs of imaginary pathways. Each pathway describes a hypothetical care pathway with slightly different characteristics compared to the alternative.

We would like you to consider each pathway’s characteristics as if you were deciding between them in real life and tell us which one you would prefer for your child or young person.

Please make sure you read each choice set carefully before ticking the option you prefer (Pathway A or Pathway B).

A pathway is described based on the characteristics listed below and each characteristic might have one of the possible values presented next to it.

| Characteristic: | Possible values for the characteristic: |
| --- | --- |
| Days missed from school in the last year  Refers to how the child's/young person’s eating disorder and the care they receive for it disrupts their education due to days missed from school. | 10 days  25 days  50 days  100 days |
| Days in hospital in the last year  Refers to the time spent in hospital or other facility by the child/young person due to their eating disorder and the care they receive for it. | 10 days  20 days  50 days |
| Functioning  Describes how the child/young person is able to function in everyday life as a consequence of their eating disorder and the care they receive for it. For example, everyday functioning may be affected due to the physical and mental health repercussions of eating disorders (e.g., stomach cramps, dizziness, fainting spells, muscle weakness, anxiety and depression). | - There is some impact on everyday functioning; some usual activities are not undertaken or require assistance. - There is severe impact on everyday functioning; usual activities are not possible, and the child/young person is unable to be independent. |
| NHS Costs  Describes how much the NHS have to pay per year for the care provided to the child/young person for their eating disorder. | £1,000  £10,000  £25,000  £40,000 |
| Interaction with peers with eating disorders  Describes the impact that the care the child/young person receives has on interacting with peers with eating disorders. This has potential negative effects, for example, by increasing exposure to negative views about body image, and competition between peers to lose weight. | - There is minor or occasional interaction with peers with eating disorders. - There is frequent or constant interaction with peers with eating disorders. |

First an example...
A person has been asked to consider the characteristics of Pathway A and Pathway B listed below. Then, they have been asked to tick the box to indicate which of the two pathways s/he would choose for their child or young person.

|  | Pathway A | Pathway B |
| --- | --- | --- |
| **Days missed from school in the last year** | 10 days | 25 days |
| **Days in hospital in the last year** | 20 days | 50 days |
| **Functioning** | There is severe impact on everyday functioning; usual activities are not possible, and the child/young person is unable to be independent. | **There is some impact on everyday functioning; some usual activities are not undertaken or require assistance.** |
| **NHS Costs** | £25,000 | £1,000 |
| Interaction with peers with eating disorders | **There is minor or occasional interaction with peers with eating disorders.** | There is frequent or constant interaction with peers with eating disorders. |

If, on balance, the person would prefer Pathway B as described rather than Pathway A, s/he would have ticked the box for Pathway B, as follows:

Pathway A Pathway B **X**

# Scenario 1

|  | Pathway A | Pathway B |
| --- | --- | --- |
| **Days missed from school in the last year** | 100 days | 25 days |
| **Days in hospital in the last year** | 50 days | 10 days |
| **Functioning** | **There is some impact on everyday functioning; some usual activities are not undertaken or require assistance.** | There is severe impact on everyday functioning; usual activities are not possible, and the child/young person is unable to be independent. |
| **NHS Costs** | £40,000 | £25,000 |
| Interaction with peers with eating disorders | **There is minor or occasional interaction with peers with eating disorders.** | There is frequent or constant interaction with peers with eating disorders. |

Which Pathway would you choose? (Tick one box only)

Pathway A Pathway B

# Scenario 2

|  | Pathway A | Pathway B |
| --- | --- | --- |
| **Days missed from school in the last year** | **10 days** | **50 days** |
| **Days in hospital in the last year** | 10 days | 50 days |
| **Functioning** | There is severe impact on everyday functioning; usual activities are not possible, and the child/young person is unable to be independent. | **There is some impact on everyday functioning; some usual activities are not undertaken or require assistance.** |
| **NHS Costs** | £40,000 | £10,000 |
| Interaction with peers with eating disorders | **There is minor or occasional interaction with peers with eating disorders.** | There is frequent or constant interaction with peers with eating disorders. |

Which Pathway would you choose? (Tick one box only)

Pathway A Pathway B

# Scenario 3

|  | Pathway A | Pathway B |
| --- | --- | --- |
| **Days missed from school in the last year** | **25 days** | **10 days** |
| **Days in hospital in the last year** | 50 days | 10 days |
| **Functioning** | There is severe impact on everyday functioning; usual activities are not possible, and the child/young person is unable to be independent. | **There is some impact on everyday functioning; some usual activities are not undertaken or require assistance.** |
| **NHS Costs** | **£1,000** | **£25,000** |
| Interaction with peers with eating disorders | There is frequent or constant interaction with peers with eating disorders. | **There is minor or occasional interaction with peers with eating disorders.** |

Which Pathway would you choose? (Tick one box only)

Pathway A Pathway B

# Scenario 4

|  | Pathway A | Pathway B |
| --- | --- | --- |
| **Days missed from school in the last year** | 100 days | 50 days |
| **Days in hospital in the last year** | **20 days** | **50 days** |
| **Functioning** | **There is some impact on everyday functioning; some usual activities are not undertaken or require assistance.** | There is severe impact on everyday functioning; usual activities are not possible, and the child/young person is unable to be independent. |
| **NHS Costs** | **£1,000** | **£25,000** |
| Interaction with peers with eating disorders | There is frequent or constant interaction with peers with eating disorders. | **There is minor or occasional interaction with peers with eating disorders.** |

Which Pathway would you choose? (Tick one box only)

Pathway A Pathway B

# Scenario 5

|  | Pathway A | Pathway B |
| --- | --- | --- |
| **Days missed from school in the last year** | 100 days | 10 days |
| **Days in hospital in the last year** | 10 days | 20 days |
| **Functioning** | There is severe impact on everyday functioning; usual activities are not possible, and the child/young person is unable to be independent. | **There is some impact on everyday functioning; some usual activities are not undertaken or require assistance.** |
| **NHS Costs** | **£10,000** | **£1,000** |
| Interaction with peers with eating disorders | There is frequent or constant interaction with peers with eating disorders. | **There is minor or occasional interaction with peers with eating disorders.** |

Which Pathway would you choose? (Tick one box only)

Pathway A Pathway B

# Scenario 6

|  | Pathway A | Pathway B |
| --- | --- | --- |
| **Days missed from school in the last year** | 100 days | 50 days |
| **Days in hospital in the last year** | **10 days** | **20 days** |
| **Functioning** | **There is some impact on everyday functioning; some usual activities are not undertaken or require assistance.** | There is severe impact on everyday functioning; usual activities are not possible, and the child/young person is unable to be independent. |
| **NHS Costs** | **£1,000** | **£25,000** |
| Interaction with peers with eating disorders | There is frequent or constant interaction with peers with eating disorders. | **There is minor or occasional interaction with peers with eating disorders.** |

Which Pathway would you choose? (Tick one box only)

Pathway A Pathway B

# Scenario 7

|  | Pathway A | Pathway B |
| --- | --- | --- |
| **Days missed from school in the last year** | **25 days** | 100 days |
| **Days in hospital in the last year** | **10 days** | **20 days** |
| **Functioning** | **There is some impact on everyday functioning; some usual activities are not undertaken or require assistance.** | There is severe impact on everyday functioning; usual activities are not possible, and the child/young person is unable to be independent. |
| **NHS Costs** | £40,000 | £10,000 |
| Interaction with peers with eating disorders | There is frequent or constant interaction with peers with eating disorders. | **There is minor or occasional interaction with peers with eating disorders.** |

Which Pathway would you choose? (Tick one box only)

Pathway A Pathway B

# Scenario 8

|  | Pathway A | Pathway B |
| --- | --- | --- |
| **Days missed from school in the last year** | **25 days** | **10 days** |
| **Days in hospital in the last year** | **20 days** | **50 days** |
| **Functioning** | **There is some impact on everyday functioning; some usual activities are not undertaken or require assistance.** | There is severe impact on everyday functioning; usual activities are not possible, and the child/young person is unable to be independent. |
| **NHS Costs** | **£25,000** | **£40,000** |
| Interaction with peers with eating disorders | **There is minor or occasional interaction with peers with eating disorders.** | There is frequent or constant interaction with peers with eating disorders. |

Which Pathway would you choose? (Tick one box only)

Pathway A Pathway B

Please tell us how easy or difficult you found this section (Section B – Discrete Choice Survey) to complete.

| □ | Very easy |
| --- | --- |
| □ | Easy |
| □ | Difficult |
| □ | Very difficult |

If you found this section of the questionnaire difficult or very difficult to complete, please tell us why in the box below.

Reason?…………………………………………………………………………………………………………………………………………………………………………………………………………………………………………………………………………………………………………………………………………………………………………………………………………………………………………………………………………………………………… …………………………………………………………………………………………………………………………………………………………………………………………………………………………………………………………………………………………………………………………………………………………………………………………………………………………………………………………………………………………………………………………………………………………………………………………………………………………………………………………………………………………………………………………………………………………………………………………………………………………………………………………………………

# Section C - Ranking

Please rank the different aspects of integration of specialized pathways for eating disorders in children and young people below in order of their importance to you with 1 being the aspect you consider most important and 5 being the aspect you consider least important.

| CHARACTERISTIC OF THE INTEGRATED PATHWAY | DEFINITION | RANK IN ORDER OF IMPORTANCE  1=most important  5= least important |
| --- | --- | --- |
| Days missed from school in the last year | Refers to how the child's/young person’s eating disorder and the care they receive for it disrupts their education due to days missed from school. |  |
| Days in hospital in the last year | Refers to the time spent in hospital or other facility by the child/young person due to their eating disorder and the care they receive for it. |  |
| Functioning | Describes how the child/young person is able to function in everyday life as a consequence of their eating disorder and the care they receive for it. For example, everyday functioning may be affected due to the physical and mental health repercussions of eating disorders (e.g., stomach cramps, dizziness, fainting spells, muscle weakness, anxiety and depression). |  |
| NHS Costs | Describes how much the NHS have to pay per year for the care provided to the child/young person for their eating disorder. |  |
| Interaction with peers with eating disorders | Describes the impact that the care the child/young person receives has on interacting with peers with eating disorders. This has potential negative effects, for example, by increasing exposure to negative views about body image, and competition between peers to lose weight. |  |

# Section D - Information about you

In this section we would like to collect some information about you.

Question 1

What is your age?

*Tick only one:*

☐18–24

☐ 25-34

☐ 35-44

☐ 45-54

☐ 55-64

☐ 65-74

☐ 75+

☐Prefer not to say

Question 2

How would you best identify yourself?

*Tick only one:*

◻ Female ◻ Male ◻ Other ◻ Prefer not to say

Question 3

Are you a single carer of one or more children or young people?

*Tick only one:*

☐Yes

☐No

☐Prefer not to say

Question 4

Which category best describes your ethnic group?

*Tick only one:*

☐White

☐Mixed or multiple ethnic groups

☐Indian

☐Pakistani

☐Bangladeshi

☐Chinese

☐Any other Asian background

☐Black, African, Black Caribbean, Black British

☐Prefer not to say

☐Other ethnic group, *provide detail below:*

Other…………………………………………………………………………………………………………………………………………………………………………………………………………………………………………………………………………………………………………………………………………………………………………………………………………………………………………………………………………………………………………………

Question 5

Which geographical region of the UK do you live in?

*Tick only one:*

| East of England  East Midlands  London  North East & Cumbria  Northern Ireland  North West of England  Scotland | South East of England  South West of England  Wales  West Midlands  Yorkshire  Prefer not to say  Other [Please specify below] |
| --- | --- |

…………………………………………………………………………………………………………………………………………………………………………………………………………………………………………………………………………………………………………………………………………………………………………………………………………………………………………………………………………………………………………………

Question 6

What is your highest level of educational qualification?

*Tick only one:*

☐No formal qualifications or equivalent

☐O level or GCSE, or equivalent

☐ONC or BTEC, or equivalent

☐A level (‘Higher’ in Scotland) or equivalent

☐Higher-education qualification below degree level or equivalent

☐Degree or higher degree or equivalent

☐Prefer not to say

Comment

Please use this box if you would like to tell us anything else about yourself or your child in relation to any aspect of care for eating disorders.

Comment…………………………………………………………………………………………………………………………………………………………………………………………………………………………………………………………………………………………………………………………………………………………………………………………………………………………………………………………………………………………………………………………………………………………

Thank you so much, you made it ☺! We hope you enjoyed taking part in this study. Your answers will be very valuable to us. If you have any comments, questions or suggestions about this survey or the CENT study in general, please write them in the box below

Comment…………………………………………………………………………………………………………………………………………………………………………………………………………………………………………………………………………………………………………………………………………………………………………………………………………………………………………………………………………………………………………………………………………………………………………………………………………………………………………………………………………………………………………………………………………………………………………………………………………………………………………………………………………………………………………………………………………………………………………………………………………………………………………………………………………………………………………………………………………………………………………………………………………………………………………………………………………………………………………………………………………………………………………………………………………………………………………………………………………………………………………………………………………………………………………………………………………………………………………………………………………………………………………………………………………………………………………………………………………………………………………………………

If you have any questions about this survey please contact:

Lina Massou: [em716@medschl.cam.ac.uk](mailto:em716@medschl.cam.ac.uk)

To find out more about the CENT study or to access the results of the study when they become available, please go to: <https://www.nuffieldtrust.org.uk/project/centralisation-of-specialist-health-care-services-a-mixed-methods-programme>

Thank you for taking part.

**Appendix II. Questionnaire for eating disorders – Block 2**

**Title of Study:** Integration of specialised services for eating disorders and functional symptoms in children and young people (CENT): a mixed methods study

**Why do we need your help?**

We are interested in informing the planned integration of physical and mental health services for children and young people with eating disorders at Cambridge Children Hospital. The information you provide will be vital in helping us understand which issues matter to you the most, and how strongly you feel about these. Based on the findings we will inform the organisation of specialist services for this condition at Cambridge and elsewhere in the NHS.

**What we would like you to do?**

In the following pages we have listed some descriptions of imaginary pathways (i.e., hypothetical care pathways), which children and young people with an eating disorder might follow. These pathways are presented in pairs, and we would like you to think about these and tell us which one sounds best to you. There is no right or wrong answer. It is possible that in some of the pairs neither of the two options exactly matches your preferences, but please don’t worry about this. Your responses should reflect your views as a parent or carer of a child or young person (regardless of any health conditions). We know some of the choices might be hard, and you might sometimes have to choose the least bad option rather than choosing one which you would actually like. Please just do your best.

# Completing the survey

Please follow the instructions and complete all of the questions in each section using the spaces provided. If you make a mistake, simply change your selection to the correct answer. You can return to a previous question using the back arrow if you want to do so.

**Taking part in this survey is voluntary. Please note you must be aged 18 years or over to take part. The research team will not be able to link your responses back to you in any way. IQVIA is the company administering the survey on behalf of the research team. Please do not put your name or address anywhere on the questionnaire.**

Finally, thank you for participating in the survey, we really do appreciate the time you give up in order to share your experiences with us and we sincerely value all the information provided.

THE CENT RESEARCH TEAM

**IMPORTANT INFORMATION**

**By completing this questionnaire, you are giving your consent for the following:**

- You are providing data to IQVIA via this questionnaire. IQVIA will not use your data for any other purpose than this specific study and will not analyse your data.
- You will not be asked to provide any information that identifies you whilst completing the questionnaire online. Once you have submitted your answers, all the responses will be anonymised by IQVIA. After this you will not be able to ask for your data to be removed because it will not be possible to work out which data are yours.
- Fully anonymous completed questionnaires will then be sent by IQVIA to University College London, UK, where they will be analysed by researchers who are part of the research team.
- Nothing will be linked to any other health care information about you that is held by any health care provider, at any point.

Your answers to the questionnaire will be processed for the purposes outlined in this notice. By clicking on the consent to participate button below you are agreeing that you have read and understood the information contained in the participant information sheet.

START THE SURVEY

# Section A - Suitability for survey

Please read the following statements carefully and tick the appropriate box.

|  | YES | NO |
| --- | --- | --- |
| I am aged 18 years or over. |  |  |
| I am parent or carer of a child or young person |  |  |
| I have read and understood the information regarding the survey. |  |  |
| I understand that by completing this questionnaire, I am giving consent for any personal details that might have been used for contact purposes, to be held by a survey company before final anonymised dataset will be sent to UCL for the purposes of this study as outlined in the information provided. |  |  |
| I am aware of who I should contact if I wish to discuss any aspect of the study. |  |  |

If you answered NO for any of the statements, you are unfortunately not eligible to take part in the survey.

# Section B – Discrete Choice Survey

In the following section you will see eight pairs of imaginary pathways. Each pathway describes a hypothetical care pathway with slightly different characteristics compared to the alternative.

We would like you to consider each pathway’s characteristics as if you were deciding between them in real life and tell us which one you would prefer for your child or young person.

Please make sure you read each choice set carefully before ticking the option you prefer (Pathway A or Pathway B).

A pathway is described based on the characteristics listed below and each characteristic might have one of the possible values presented next to it.

| Characteristic: | Possible values for the characteristic: |
| --- | --- |
| Days missed from school in the last year  Refers to how the child's/young person’s eating disorder and the care they receive for it disrupts their education due to days missed from school. | 10 days  25 days  50 days  100 days |
| Days in hospital in the last year  Refers to the time spent in hospital or other facility by the child/young person due to their eating disorder and the care they receive for it. | 10 days  20 days  50 days |
| Functioning  Describes how the child/young person is able to function in everyday life as a consequence of their eating disorder and the care they receive for it. For example, everyday functioning may be affected due to the physical and mental health repercussions of eating disorders (e.g., stomach cramps, dizziness, fainting spells, muscle weakness, anxiety and depression). | - There is some impact on everyday functioning; some usual activities are not undertaken or require assistance. - There is severe impact on everyday functioning; usual activities are not possible, and the child/young person is unable to be independent. |
| NHS Costs  Describes how much the NHS have to pay per year for the care provided to the child/young person for their eating disorder. | £1,000  £10,000  £25,000  £40,000 |
| Interaction with peers with eating disorders  Describes the impact that the care the child/young person receives has on interacting with peers with eating disorders. This has potential negative effects, for example, by increasing exposure to negative views about body image, and competition between peers to lose weight. | - There is minor or occasional interaction with peers with eating disorders. - There is frequent or constant interaction with peers with eating disorders. |

First an example...
A person has been asked to consider the characteristics of Pathway A and Pathway B listed below. Then, they have been asked to tick the box to indicate which of the two pathways s/he would choose for their child or young person.

|  | Pathway A | Pathway B |
| --- | --- | --- |
| **Days missed from school in the last year** | 10 days | 25 days |
| **Days in hospital in the last year** | 20 days | 50 days |
| **Functioning** | There is severe impact on everyday functioning; usual activities are not possible, and the child/young person is unable to be independent. | **There is some impact on everyday functioning; some usual activities are not undertaken or require assistance.** |
| **NHS Costs** | £25,000 | £1,000 |
| Interaction with peers with eating disorders | **There is minor or occasional interaction with peers with eating disorders.** | There is frequent or constant interaction with peers with eating disorders. |

If, on balance, the person would prefer Pathway B as described rather than Pathway A, s/he would have ticked the box for Pathway B, as follows:

Pathway A Pathway B **X**

# Scenario 1

|  | Pathway A | Pathway B |
| --- | --- | --- |
| **Days missed from school in the last year** | 25 days | 50 days |
| **Days in hospital in the last year** | 20 days | 10 days |
| **Functioning** | **There is some impact on everyday functioning; some usual activities are not undertaken or require assistance.** | There is severe impact on everyday functioning; usual activities are not possible, and the child/young person is unable to be independent. |
| **NHS Costs** | £40,000 | £1,000 |
| Interaction with peers with eating disorders | There is frequent or constant interaction with peers with eating disorders. | There is minor or occasional interaction with peers with eating disorders. |

Which Pathway would you choose? (Tick one box only)

Pathway A Pathway B

# Scenario 2

|  | Pathway A | Pathway B |
| --- | --- | --- |
| **Days missed from school in the last year** | **25 days** | **50 days** |
| **Days in hospital in the last year** | 50 days | 10 days |
| **Functioning** | There is some impact on everyday functioning; some usual activities are not undertaken or require assistance. | **There is severe impact on everyday functioning; usual activities are not possible, and the child/young person is unable to be independent.** |
| **NHS Costs** | £1,000 | £40,000 |
| Interaction with peers with eating disorders | **There is minor or occasional interaction with peers with eating disorders.** | There is frequent or constant interaction with peers with eating disorders. |

Which Pathway would you choose? (Tick one box only)

Pathway A Pathway B

# Scenario 3

|  | Pathway A | Pathway B |
| --- | --- | --- |
| **Days missed from school in the last year** | **25 days** | **50 days** |
| **Days in hospital in the last year** | 10 days | 50 days |
| **Functioning** | There is severe impact on everyday functioning; usual activities are not possible, and the child/young person is unable to be independent. | **There is some impact on everyday functioning; some usual activities are not undertaken or require assistance.** |
| **NHS Costs** | **£10,000** | **£25,000** |
| Interaction with peers with eating disorders | **There is minor or occasional interaction with peers with eating disorders.** | **There is frequent or constant interaction with peers with eating disorders.** |

Which Pathway would you choose? (Tick one box only)

Pathway A Pathway B

# Scenario 4

|  | Pathway A | Pathway B |
| --- | --- | --- |
| **Days missed from school in the last year** | 100 days | 10 days |
| **Days in hospital in the last year** | **10 days** | **20 days** |
| **Functioning** | **There is some impact on everyday functioning; some usual activities are not undertaken or require assistance.** | There is severe impact on everyday functioning; usual activities are not possible, and the child/young person is unable to be independent. |
| **NHS Costs** | **£25,000** | **£10,000** |
| Interaction with peers with eating disorders | There is minor or occasional interaction with peers with eating disorders. | **There is frequent or constant interaction with peers with eating disorders.** |

Which Pathway would you choose? (Tick one box only)

Pathway A Pathway B

# Scenario 5

|  | Pathway A | Pathway B |
| --- | --- | --- |
| **Days missed from school in the last year** | 50 days | 10 days |
| **Days in hospital in the last year** | 20 days | 50 days |
| **Functioning** | There is some impact on everyday functioning; some usual activities are not undertaken or require assistance. | **There is severe impact on everyday functioning; usual activities are not possible, and the child/young person is unable to be independent.** |
| **NHS Costs** | **£40,000** | **£25,000** |
| Interaction with peers with eating disorders | There is minor or occasional interaction with peers with eating disorders. | **There is frequent or constant interaction with peers with eating disorders.** |

Which Pathway would you choose? (Tick one box only)

Pathway A Pathway B

# Scenario 6

|  | Pathway A | Pathway B |
| --- | --- | --- |
| **Days missed from school in the last year** | 50 days | 10 days |
| **Days in hospital in the last year** | **20 days** | **50 days** |
| **Functioning** | **There is severe impact on everyday functioning; usual activities are not possible, and the child/young person is unable to be independent.** | There is some impact on everyday functioning; some usual activities are not undertaken or require assistance. |
| **NHS Costs** | **£1,000** | **£10,000** |
| Interaction with peers with eating disorders | There is frequent or constant interaction with peers with eating disorders. | **There is minor or occasional interaction with peers with eating disorders.** |

Which Pathway would you choose? (Tick one box only)

Pathway A Pathway B

# Scenario 7

|  | Pathway A | Pathway B |
| --- | --- | --- |
| **Days missed from school in the last year** | **10 days** | **25 days** |
| **Days in hospital in the last year** | **20 days** | **50 days** |
| **Functioning** | **There is some impact on everyday functioning; some usual activities are not undertaken or require assistance.** | There is severe impact on everyday functioning; usual activities are not possible, and the child/young person is unable to be independent. |
| **NHS Costs** | £10,000 | £40,000 |
| Interaction with peers with eating disorders | There is frequent or constant interaction with peers with eating disorders. | **There is minor or occasional interaction with peers with eating disorders.** |

Which Pathway would you choose? (Tick one box only)

Pathway A Pathway B

# Scenario 8

|  | Pathway A | Pathway B |
| --- | --- | --- |
| **Days missed from school in the last year** | **50 days** | **100 days** |
| **Days in hospital in the last year** | **10 days** | **20 days** |
| **Functioning** | **There is some impact on everyday functioning; some usual activities are not undertaken or require assistance.** | There is severe impact on everyday functioning; usual activities are not possible, and the child/young person is unable to be independent. |
| **NHS Costs** | **£10,000** | **£25,000** |
| Interaction with peers with eating disorders | **There is minor or occasional interaction with peers with eating disorders.** | There is frequent or constant interaction with peers with eating disorders. |

Which Pathway would you choose? (Tick one box only)

Pathway A Pathway B

Please tell us how easy or difficult you found this section (Section B – Discrete Choice Survey) to complete.

| □ | Very easy |
| --- | --- |
| □ | Easy |
| □ | Difficult |
| □ | Very difficult |

If you found this section of the questionnaire difficult or very difficult to complete, please tell us why in the box below.

Reason?…………………………………………………………………………………………………………………………………………………………………………………………………………………………………………………………………………………………………………………………………………………………………………………………………………………………………………………………………………………………………… …………………………………………………………………………………………………………………………………………………………………………………………………………………………………………………………………………………………………………………………………………………………………………………………………………………………………………………………………………………………………………………………………………………………………………………………………………………………………………………………………………………………………………………………………………………………………………………………………………………………………………………………………………

# Section C - Ranking

Please rank the different aspects of integration of specialized pathways for eating disorders in children and young people below in order of their importance to you with 1 being the aspect you consider most important and 5 being the aspect you consider least important.

| CHARACTERISTIC OF THE INTEGRATED PATHWAY | DEFINITION | RANK IN ORDER OF IMPORTANCE  1=most important  5= least important |
| --- | --- | --- |
| Days missed from school in the last year | Refers to how the child's/young person’s eating disorder and the care they receive for it disrupts their education due to days missed from school. |  |
| Days in hospital in the last year | Refers to the time spent in hospital or other facility by the child/young person due to their eating disorder and the care they receive for it. |  |
| Functioning | Describes how the child/young person is able to function in everyday life as a consequence of their eating disorder and the care they receive for it. For example, everyday functioning may be affected due to the physical and mental health repercussions of eating disorders (e.g., stomach cramps, dizziness, fainting spells, muscle weakness, anxiety and depression). |  |
| NHS Costs | Describes how much the NHS have to pay per year for the care provided to the child/young person for their eating disorder. |  |
| Interaction with peers with eating disorders | Describes the impact that the care the child/young person receives has on interacting with peers with eating disorders. This has potential negative effects, for example, by increasing exposure to negative views about body image, and competition between peers to lose weight. |  |

# Section D - Information about you

In this section we would like to collect some information about you.

Question 1

What is your age?

*Tick only one:*

☐18–24

☐ 25-34

☐ 35-44

☐ 45-54

☐ 55-64

☐ 65-74

☐ 75+

☐Prefer not to say

Question 2

How would you best identify yourself?

*Tick only one:*

◻ Female ◻ Male ◻ Other ◻ Prefer not to say

Question 3

Are you a single carer of one or more children or young people?

*Tick only one:*

☐Yes

☐No

☐Prefer not to say

Question 4

Which category best describes your ethnic group?

*Tick only one:*

☐White

☐Mixed or multiple ethnic groups

☐Indian

☐Pakistani

☐Bangladeshi

☐Chinese

☐Any other Asian background

☐Black, African, Black Caribbean, Black British

☐Prefer not to say

☐Other ethnic group, *provide detail below:*

Other…………………………………………………………………………………………………………………………………………………………………………………………………………………………………………………………………………………………………………………………………………………………………………………………………………………………………………………………………………………………………………………

Question 5

Which geographical region of the UK do you live in?

*Tick only one:*

| East of England  East Midlands  London  North East & Cumbria  Northern Ireland  North West of England  Scotland | South East of England  South West of England  Wales  West Midlands  Yorkshire  Prefer not to say  Other [Please specify below] |
| --- | --- |

…………………………………………………………………………………………………………………………………………………………………………………………………………………………………………………………………………………………………………………………………………………………………………………………………………………………………………………………………………………………………………………

Question 6

What is your highest level of educational qualification?

*Tick only one:*

☐No formal qualifications or equivalent

☐O level or GCSE, or equivalent

☐ONC or BTEC, or equivalent

☐A level (‘Higher’ in Scotland) or equivalent

☐Higher-education qualification below degree level or equivalent

☐Degree or higher degree or equivalent

☐Prefer not to say

Comment

Please use this box if you would like to tell us anything else about yourself or your child in relation to any aspect of care for eating disorders.

Comment…………………………………………………………………………………………………………………………………………………………………………………………………………………………………………………………………………………………………………………………………………………………………………………………………………………………………………………………………………………………………………………………………………………………

Thank you so much, you made it ☺! We hope you enjoyed taking part in this study. Your answers will be very valuable to us. If you have any comments, questions or suggestions about this survey or the CENT study in general, please write them in the box below

Comment…………………………………………………………………………………………………………………………………………………………………………………………………………………………………………………………………………………………………………………………………………………………………………………………………………………………………………………………………………………………………………………………………………………………………………………………………………………………………………………………………………………………………………………………………………………………………………………………………………………………………………………………………………………………………………………………………………………………………………………………………………………………………………………………………………………………………………………………………………………………………………………………………………………………………………………………………………………………………………………………………………………………………………………………………………………………………………………………………………………………………………………………………………………………………………………………………………………………………………………………………………………………………………………………………………………………………………………………………………………………………………………………

If you have any questions about this survey please contact:

Lina Massou: em716@medschl.cam.ac.uk

To find out more about the CENT study or to access the results of the study when they become available, please go to: <https://www.nuffieldtrust.org.uk/project/centralisation-of-specialist-health-care-services-a-mixed-methods-programme>

Thank you for taking part.

**Appendix III. Questionnaire for functional symptom disorders – Block 1**

**Title of Study:** Integration of specialised services for eating disorders and functional symptoms in children and young people (CENT): a mixed methods study

**Why do we need your help?**

We are interested in informing the planned integration of physical and mental health services for children and young people with functional symptoms at Cambridge Children Hospital. The information you provide will be vital in helping us understand which issues matter to you the most, and how strongly you feel about these. Based on the findings we will inform the organisation of specialist services for this condition at Cambridge and elsewhere in the NHS.

**What we would like you to do?**

In the following pages we have listed some descriptions of imaginary pathways (i.e., hypothetical care pathways), which children and young people with a functional symptom might follow. These pathways are presented in pairs, and we would like you to think about these and tell us which one sounds best to you. There is no right or wrong answer. It is possible that in some of the pairs neither of the two options exactly matches your preferences, but please don’t worry about this. Your responses should reflect your views as a parent or carer of a child or young person (regardless of any health conditions). We know some of the choices might be hard, and you might sometimes have to choose the least bad option rather than choosing one which you would actually like. Please just do your best.

# **Completing the survey**

Please follow the instructions and complete all of the questions in each section using the spaces provided. If you make a mistake, simply change your selection to the correct answer. You can return to a previous question using the back arrow if you want to do so.

**Taking part in this survey is voluntary. Please note you must be aged 18 years or over to take part. The research team will not be able to link your responses back to you in any way. IQVIA is the company administering the survey on behalf of the research team. Please do not put your name or address anywhere on the questionnaire.**

Finally, thank you for participating in the survey, we really do appreciate the time you give up in order to share your experiences with us and we sincerely value all the information provided.

THE CENT RESEARCH TEAM

**IMPORTANT INFORMATION**

**By completing this questionnaire, you are giving your consent for the following:**

- You are providing data to IQVIA via this questionnaire. IQVIA will not use your data for any other purpose than this specific study and will not analyse your data.
- You will not be asked to provide any information that identifies you whilst completing the questionnaire online. Once you have submitted your answers, all the responses will be anonymised by IQVIA. After this you will not be able to ask for your data to be removed because it will not be possible to work out which data are yours.
- Fully anonymous completed questionnaires will then be sent by IQVIA to University College London, UK, where they will be analysed by researchers who are part of the research team.
- Nothing will be linked to any other health care information about you that is held by any health care provider, at any point.

Your answers to the questionnaire will be processed for the purposes outlined in this notice. By clicking on the consent to participate button below you are agreeing that you have read and understood the information contained in the participant information sheet.

START THE SURVEY

# Section A - Suitability for survey

Please read the following statements carefully and tick the appropriate box.

|  | YES | NO |
| --- | --- | --- |
| I am aged 18 years or over. |  |  |
| I am parent or carer of a child or young person |  |  |
| I have read and understood the information regarding the survey. |  |  |
| I understand that by completing this questionnaire, I am giving consent for any personal details that might have been used for contact purposes, to be held by a survey company before final anonymised dataset will be sent to UCL for the purposes of this study as outlined in the information provided. |  |  |
| I am aware of who I should contact if I wish to discuss any aspect of the study. |  |  |

If you answered NO for any of the statements, you are unfortunately not eligible to take part in the survey.

# Section B – Discrete Choice Survey

In the following section you will see eight pairs of imaginary pathways. Each pathway describes a hypothetical care pathway with slightly different characteristics compared to the alternative.

We would like you to consider each pathway’s characteristics as if you were deciding between them in real life and tell us which one you would prefer for your child or young person.

Please make sure you read each choice set carefully before ticking the option you prefer (Pathway A or Pathway B).

A pathway is described based on the characteristics listed below and each characteristic might have one of the possible values presented next to it.

| Characteristic | Possible values |
| --- | --- |
| Reservations about seeing a mental health practitioner  Refers to extent of reservations about the child/young person seeing a mental health practitioner (e.g., a psychologist or psychiatrist) and the possibility of them requiring psychological/ psychiatric treatment. These reservations might arise from the fear of stigma and prejudice regarding mental health conditions, and the concerns that the symptoms the child/young person experience are not “real”. | - I have some reservations about the child/young person seeing a mental health practitioner. - I have major reservations about the child/young person seeing a mental health practitioner. |
| Time to diagnosis  Refers to the length of time needed from initial contact about the child’s/young person’s symptoms with primary care until a final diagnosis is obtained. This does not include potential diagnoses that are explored across the diagnostic pathway. | - 3 months since the initial contact - 6 months since the initial contact - 12 months since the initial contact - 18 months since the initial contact - 24 months since the initial contact |
| NHS costs of diagnosis  Refers to the costs borne by the NHS to reach a diagnosis for the child/young person, for example arising from the number of clinic visits and diagnostic tests needed to reach a diagnosis | - £1,000 - £5,000 - £10,000 - £15,000 |
| Days missed from school while obtaining a diagnosis  Refers to how the child's/young person’s symptoms and the health care contacts they receive for these disrupts their education due to days missed from school. This covers the time from the child/young person first experiencing their symptoms until a final diagnosis is obtained | - 10 days - 20 days - 30 days - 50 days |

**First an example...**

A person has been asked to consider the characteristics of Pathway A and Pathway B listed below. Then, they have been asked to tick the box to indicate which of the two pathways s/he would choose for their child or young person.

|  | Pathway A | Pathway B |
| --- | --- | --- |
| **Reservations about seeing a mental health practitioner** | I have some reservations about the child/young person seeing a mental health practitioner. | I have major reservations about the child/young person seeing a mental health practitioner. |
| **Time to diagnosis** | 18 months since the initial contact | 3 months since the initial contact |
| **NHS costs of diagnosis** | £5,000 | £1,000 |
| **Days missed from school while obtaining a diagnosis** | 50 days | 10 days |

If, on balance, the person would prefer Pathway B as described rather than Pathway A, s/he would have ticked the box for Pathway B, as follows:

Pathway A Pathway B **X**

# Scenario 1

|  | Pathway A | Pathway B |
| --- | --- | --- |
| **Reservations about seeing a mental health practitioner** | I have some reservations about the child/young person seeing a mental health practitioner. | I have major reservations about the child/young person seeing a mental health practitioner. |
| Time to diagnosis | **6 months since the initial contact** | **3 months since the initial contact** |
| **NHS costs of diagnosis** | £15,000 | £5,000 |
| **Days missed from school while obtaining a diagnosis** | **30 days** | 50 days |

Which Pathway would you choose? (Tick one box only)

Pathway A Pathway B

# Scenario 2

|  | Pathway A | Pathway B |
| --- | --- | --- |
| **Reservations about seeing a mental health practitioner** | I have major reservations about the child/young person seeing a mental health practitioner. | **I have some reservations about the child/young person seeing a mental health practitioner.** |
| Time to diagnosis | **18 months since the initial contact** | **6 months since the initial contact** |
| **NHS costs of diagnosis** | **£10,000** | **£5,000** |
| **Days missed from school while obtaining a diagnosis** | **10 days** | 50 days |

Which Pathway would you choose? (Tick one box only)

Pathway A Pathway B

# Scenario 3

|  | Pathway A | Pathway B |
| --- | --- | --- |
| **Reservations about seeing a mental health practitioner** | I have major reservations about the child/young person seeing a mental health practitioner. | I have some reservations about the child/young person seeing a mental health practitioner. |
| Time to diagnosis | **3 months since the initial contact** | **24 months since the initial contact** |
| **NHS costs of diagnosis** | **£5,000** | **£1,000** |
| **Days missed from school while obtaining a diagnosis** | **30 days** | **10 days** |

Which Pathway would you choose? (Tick one box only)

Pathway A Pathway B

# Scenario 4

|  | Pathway A | Pathway B |
| --- | --- | --- |
| **Reservations about seeing a mental health practitioner** | **I have some reservations about the child/young person seeing a mental health practitioner.** | I have major reservations about the child/young person seeing a mental health practitioner. |
| Time to diagnosis | **18 months since the initial contact** | 1. **months since the initial contact** |
| **NHS costs of diagnosis** | **£10,000** | **£15,000** |
| **Days missed from school while obtaining a diagnosis** | 50 days | 20 days |

Which Pathway would you choose? (Tick one box only)

Pathway A Pathway B

# Scenario 5

|  | Pathway A | Pathway B |
| --- | --- | --- |
| **Reservations about seeing a mental health practitioner** | I have some reservations about the child/young person seeing a mental health practitioner. | I have major reservations about the child/young person seeing a mental health practitioner. |
| Time to diagnosis | **6 months since the initial contact** | **24 months since the initial contact** |
| **NHS costs of diagnosis** | **£10,000** | **£1,000** |
| **Days missed from school while obtaining a diagnosis** | **20 days** | **30 days** |

Which Pathway would you choose? (Tick one box only)

Pathway A Pathway B

# Scenario 6

|  | Pathway A | Pathway B |
| --- | --- | --- |
| **Reservations about seeing a mental health practitioner** | I have some reservations about the child/young person seeing a mental health practitioner. | I have major reservations about the child/young person seeing a mental health practitioner. |
| Time to diagnosis | **12 months since the initial contact** | **18 months since the initial contact** |
| **NHS costs of diagnosis** | **£10,000** | £15,000 |
| **Days missed from school while obtaining a diagnosis** | **30 days** | 20 days |

Which Pathway would you choose? (Tick one box only)

Pathway A Pathway B

# Scenario 7

|  | Pathway A | Pathway B |
| --- | --- | --- |
| **Reservations about seeing a mental health practitioner** | I have major reservations about the child/young person seeing a mental health practitioner. | **I have some reservations about the child/young person seeing a mental health practitioner.** |
| Time to diagnosis | **6 months since the initial contact** | **3 months since the initial contact** |
| **NHS costs of diagnosis** | £15,000 | £1,000 |
| **Days missed from school while obtaining a diagnosis** | 10 days | 30 days |

Which Pathway would you choose? (Tick one box only)

Pathway A Pathway B

# Scenario 8

|  | Pathway A | Pathway B |
| --- | --- | --- |
| **Reservations about seeing a mental health practitioner** | **I have some reservations about the child/young person seeing a mental health practitioner.** | I have major reservations about the child/young person seeing a mental health practitioner. |
| Time to diagnosis | **12 months since the initial contact** | **24 months since the initial contact** |
| **NHS costs of diagnosis** | £1,000 | £10,000 |
| **Days missed from school while obtaining a diagnosis** | 50 days | 30 days |

Which Pathway would you choose? (Tick one box only)

Pathway A Pathway B

Please tell us how easy or difficult you found this section (Section B – Discrete Choice Survey) to complete.

| □ | Very easy |
| --- | --- |
| □ | Easy |
| □ | Difficult |
| □ | Very difficult |

If you found this section of the questionnaire difficult or very difficult to complete, please tell us why in the box below.

Reason?…………………………………………………………………………………………………………………………………………………………………………………………………………………………………………………………………………………………………………………………………………………………………………………………………………………………………………………………………………………………………… …………………………………………………………………………………………………………………………………………………………………………………………………………………………………………………………………………………………………………………………………………………………………………………………………………………………………………………………………………………………………………………………………………………………………………………………………………………………………………………………………………………………………………………………………………………………………………………………………………………………………………………………………………

# Section C - Ranking

Please rank the different aspects of integration of specialised services for functional symptoms in children and young people below in order of their importance to you with 1 being the aspect you consider most important and 4 being the aspect you consider least important.

| ASPECTS OF SPECIALIZED SERVICES | DEFINITION | RANK IN ORDER OF IMPORTANCE  1=most important  4= least important |
| --- | --- | --- |
| Reservations about seeing a mental health practitioner | Refers to extent of reservations about the child/young person seeing a mental health practitioner (e.g., a psychologist or psychiatrist) and the possibility of them requiring psychological/ psychiatric treatment. These reservations might arise from the fear of stigma and prejudice regarding mental health conditions, and the concerns that the symptoms the child/young person experience are not “real”. |  |
| Time to diagnosis | Refers to the length of time needed from initial contact about the child’s/young person’s symptoms with primary care until a final diagnosis is obtained. This does not include potential diagnoses that are explored across the diagnostic pathway. |  |
| NHS costs of diagnosis | Refers to the costs borne by the NHS to reach a diagnosis for the child/young person, for example arising from the number of clinic visits and diagnostic tests needed to reach a diagnosis |  |
| Days missed from school till the final diagnosis | Refers to the length of time needed from initial contact about the child’s/young person’s symptoms with primary care until a final diagnosis is obtained. This does not include potential diagnoses that are explored across the diagnostic pathway. |  |

# Section D - Information about you

In this section we would like to collect some information about you.

**Question 1**

What is your age?

*Tick only one:*

☐18–24

☐ 25-34

☐ 35-44

☐ 45-54

☐ 55-64

☐ 65-74

☐ 75+

☐Prefer not to say

Question 2

How would you best identify yourself?

*Tick only one:*

◻ Female ◻ Male ◻ Other ◻ Prefer not to say

**Question 3**

Are you a single carer of one or more children or young people?

*Tick only one:*

☐Yes

☐No

☐Prefer not to say

**Question 4**

Which category best describes your ethnic group?

*Tick only one:*

☐White

☐Mixed or multiple ethnic groups

☐Indian

☐Pakistani

☐Bangladeshi

☐Chinese

☐Any other Asian background

☐Black, African, Black Caribbean, Black British

☐Prefer not to say

☐Other ethnic group, *provide detail below:*

Other…………………………………………………………………………………………………………………………………………………………………………………………………………………………………………………………………………………………………………………………………………………………………………………………………………………………………………………………………………………………………………………

**Question 5**

Which geographical region of the UK do you live in?

*Tick only one:*

| East of England  East Midlands  London  North East & Cumbria  Northern Ireland  North West of England  Scotland | South East of England  South West of England  Wales  West Midlands  Yorkshire  Prefer not to say  Other [Please specify below] |
| --- | --- |

…………………………………………………………………………………………………………………………………………………………………………………………………………………………………………………………………………………………………………………………………………………………………………………………………………………………………………………………………………………………………………………

**Question 6**

What is your highest level of educational qualification?

*Tick only one:*

☐No formal qualifications or equivalent

☐O level or GCSE, or equivalent

☐ONC or BTEC, or equivalent

☐A level (‘Higher’ in Scotland) or equivalent

☐Higher-education qualification below degree level or equivalent

☐Degree or higher degree or equivalent

☐Prefer not to say

Comment

Please use this box if you would like to tell us anything else about yourself in relation to any aspect of care for functional symptoms.

Comment…………………………………………………………………………………………………………………………………………………………………………………………………………………………………………………………………………………………………………………………………………………………………………………………………………………………………………………………………………………………………………………………………………………………

Thank you so much, you made it ☺! We hope you enjoyed taking part in this study. Your answers will be very valuable to us. If you have any comments, questions or suggestions about this survey or the CENT study in general, please write them in the box below

Comment…………………………………………………………………………………………………………………………………………………………………………………………………………………………………………………………………………………………………………………………………………………………………………………………………………………………………………………………………………………………………………………………………………………………………………………………………………………………………………………………………………………………………………………………………………………………………………………………………………………………………………………………………………………………………………………………………………………………………………………………………………………………………………………………………………………………………………………………………………………………………………………………………………………………………………………………………………………………………………………………………………………………………………………………………………………………………………………………………………………………………………………………………………………………………………………………………………………………………………………………………………………………………………………………………………………………………………………………………………………………………………………………

If you have any questions about this survey please contact:

Lina Massou: em716@medschl.cam.ac.uk

To find out more about the CENT study or to access the results of the study when they become available, please go to: <https://www.nuffieldtrust.org.uk/project/centralisation-of-specialist-health-care-services-a-mixed-methods-programme>

Thank you for taking part.

**Appendix IV. Questionnaire for functional symptom disorders – Block 2**

**Title of Study:** Integration of specialised services for eating disorders and functional symptoms in children and young people (CENT): a mixed methods study

**Why do we need your help?**

We are interested in informing the planned integration of physical and mental health services for children and young people with functional symptoms at Cambridge Children Hospital. The information you provide will be vital in helping us understand which issues matter to you the most, and how strongly you feel about these. Based on the findings we will inform the organisation of specialist services for this condition at Cambridge and elsewhere in the NHS.

**What we would like you to do?**

In the following pages we have listed some descriptions of imaginary pathways (i.e., hypothetical care pathways), which children and young people with a functional symptom might follow. These pathways are presented in pairs, and we would like you to think about these and tell us which one sounds best to you. There is no right or wrong answer. It is possible that in some of the pairs neither of the two options exactly matches your preferences, but please don’t worry about this. Your responses should reflect your views as a parent or carer of a child or young person (regardless of any health conditions). We know some of the choices might be hard, and you might sometimes have to choose the least bad option rather than choosing one which you would actually like. Please just do your best.

# **Completing the survey**

Please follow the instructions and complete all of the questions in each section using the spaces provided. If you make a mistake, simply change your selection to the correct answer. You can return to a previous question using the back arrow if you want to do so.

**Taking part in this survey is voluntary. Please note you must be aged 18 years or over to take part. The research team will not be able to link your responses back to you in any way. IQVIA is the company administering the survey on behalf of the research team. Please do not put your name or address anywhere on the questionnaire.**

Finally, thank you for participating in the survey, we really do appreciate the time you give up in order to share your experiences with us and we sincerely value all the information provided.

THE CENT RESEARCH TEAM

**IMPORTANT INFORMATION**

**By completing this questionnaire, you are giving your consent for the following:**

- You are providing data to IQVIA via this questionnaire. IQVIA will not use your data for any other purpose than this specific study and will not analyse your data.
- You will not be asked to provide any information that identifies you whilst completing the questionnaire online. Once you have submitted your answers, all the responses will be anonymised by IQVIA. After this you will not be able to ask for your data to be removed because it will not be possible to work out which data are yours.
- Fully anonymous completed questionnaires will then be sent by IQVIA to University College London, UK, where they will be analysed by researchers who are part of the research team.
- Nothing will be linked to any other health care information about you that is held by any health care provider, at any point.

Your answers to the questionnaire will be processed for the purposes outlined in this notice. By clicking on the consent to participate button below you are agreeing that you have read and understood the information contained in the participant information sheet.

START THE SURVEY

# Section A - Suitability for survey

Please read the following statements carefully and tick the appropriate box.

|  | YES | NO |
| --- | --- | --- |
| I am aged 18 years or over. |  |  |
| I am parent or carer of a child or young person |  |  |
| I have read and understood the information regarding the survey. |  |  |
| I understand that by completing this questionnaire, I am giving consent for any personal details that might have been used for contact purposes, to be held by a survey company before final anonymised dataset will be sent to UCL for the purposes of this study as outlined in the information provided. |  |  |
| I am aware of who I should contact if I wish to discuss any aspect of the study. |  |  |

If you answered NO for any of the statements, you are unfortunately not eligible to take part in the survey.

# Section B – Discrete Choice Survey

In the following section you will see eight pairs of imaginary pathways. Each pathway describes a hypothetical care pathway with slightly different characteristics compared to the alternative.

We would like you to consider each pathway’s characteristics as if you were deciding between them in real life and tell us which one you would prefer for your child or young person.

Please make sure you read each choice set carefully before ticking the option you prefer (Pathway A or Pathway B).

A pathway is described based on the characteristics listed below and each characteristic might have one of the possible values presented next to it.

| Characteristic | Possible values |
| --- | --- |
| Reservations about seeing a mental health practitioner  Refers to extent of reservations about the child/young person seeing a mental health practitioner (e.g., a psychologist or psychiatrist) and the possibility of them requiring psychological/ psychiatric treatment. These reservations might arise from the fear of stigma and prejudice regarding mental health conditions, and the concerns that the symptoms the child/young person experience are not “real”. | - I have some reservations about the child/young person seeing a mental health practitioner. - I have major reservations about the child/young person seeing a mental health practitioner. |
| Time to diagnosis  Refers to the length of time needed from initial contact about the child’s/young person’s symptoms with primary care until a final diagnosis is obtained. This does not include potential diagnoses that are explored across the diagnostic pathway. | - 3 months since the initial contact - 6 months since the initial contact - 12 months since the initial contact - 18 months since the initial contact - 24 months since the initial contact |
| NHS costs of diagnosis  Refers to the costs borne by the NHS to reach a diagnosis for the child/young person, for example arising from the number of clinic visits and diagnostic tests needed to reach a diagnosis | - £1,000 - £5,000 - £10,000 - £15,000 |
| Days missed from school while obtaining a diagnosis  Refers to how the child's/young person’s symptoms and the health care contacts they receive for these disrupts their education due to days missed from school. This covers the time from the child/young person first experiencing their symptoms until a final diagnosis is obtained | - 10 days - 20 days - 30 days - 50 days |

**First an example...**

A person has been asked to consider the characteristics of Pathway A and Pathway B listed below. Then, they have been asked to tick the box to indicate which of the two pathways s/he would choose for their child or young person.

|  | Pathway A | Pathway B |
| --- | --- | --- |
| **Reservations about seeing a mental health practitioner** | I have some reservations about the child/young person seeing a mental health practitioner. | I have major reservations about the child/young person seeing a mental health practitioner. |
| **Time to diagnosis** | 18 months since the initial contact | 3 months since the initial contact |
| **NHS costs of diagnosis** | £5,000 | £1,000 |
| **Days missed from school while obtaining a diagnosis** | 50 days | 10 days |

If, on balance, the person would prefer Pathway B as described rather than Pathway A, s/he would have ticked the box for Pathway B, as follows:

Pathway A Pathway B **X**

# Scenario 1

|  | Pathway A | Pathway B |
| --- | --- | --- |
| **Reservations about seeing a mental health practitioner** | I have major reservations about the child/young person seeing a mental health practitioner. | I have some reservations about the child/young person seeing a mental health practitioner. |
| Time to diagnosis | **12 months since the initial contact** | **24 months since the initial contact** |
| **NHS costs of diagnosis** | £15,000 | £5,000 |
| **Days missed from school while obtaining a diagnosis** | **30 days** | 20 days |

Which Pathway would you choose? (Tick one box only)

Pathway A Pathway B

# Scenario 2

|  | Pathway A | Pathway B |
| --- | --- | --- |
| **Reservations about seeing a mental health practitioner** | I have some reservations about the child/young person seeing a mental health practitioner. | **I have major reservations about the child/young person seeing a mental health practitioner.** |
| Time to diagnosis | 3 months since the initial contact | 18 months since the initial contact |
| **NHS costs of diagnosis** | **£15,000** | **£1,000** |
| **Days missed from school while obtaining a diagnosis** | 10 days | 20 days |

Which Pathway would you choose? (Tick one box only)

Pathway A Pathway B

# Scenario 3

|  | Pathway A | Pathway B |
| --- | --- | --- |
| **Reservations about seeing a mental health practitioner** | I have some reservations about the child/young person seeing a mental health practitioner. | I have major reservations about the child/young person seeing a mental health practitioner. |
| Time to diagnosis | **18 months since the initial contact** | **12 months since the initial contact** |
| **NHS costs of diagnosis** | **£15,000** | **£10,000** |
| **Days missed from school while obtaining a diagnosis** | 30 days | 20 days |

Which Pathway would you choose? (Tick one box only)

Pathway A Pathway B

# Scenario 4

|  | Pathway A | Pathway B |
| --- | --- | --- |
| **Reservations about seeing a mental health practitioner** | **I have some reservations about the child/young person seeing a mental health practitioner.** | I have major reservations about the child/young person seeing a mental health practitioner. |
| Time to diagnosis | **18 months since the initial contact** | **6 months since the initial contact** |
| **NHS costs of diagnosis** | **£5,000** | **£1,000** |
| **Days missed from school while obtaining a diagnosis** | 10 days | 50 days |

Which Pathway would you choose? (Tick one box only)

Pathway A Pathway B

# Scenario 5

|  | Pathway A | Pathway B |
| --- | --- | --- |
| **Reservations about seeing a mental health practitioner** | I have some reservations about the child/young person seeing a mental health practitioner. | I have major reservations about the child/young person seeing a mental health practitioner. |
| Time to diagnosis | **6 months since the initial contact** | **12 months since the initial contact** |
| **NHS costs of diagnosis** | **£1,000** | **£15,000** |
| **Days missed from school while obtaining a diagnosis** | 10 days | 50 days |

Which Pathway would you choose? (Tick one box only)

Pathway A Pathway B

# Scenario 6

|  | Pathway A | Pathway B |
| --- | --- | --- |
| **Reservations about seeing a mental health practitioner** | I have some reservations about the child/young person seeing a mental health practitioner. | I have major reservations about the child/young person seeing a mental health practitioner. |
| Time to diagnosis | **3 months since the initial contact** | **6 months since the initial contact** |
| **NHS costs of diagnosis** | **£15,000** | £5,000 |
| **Days missed from school while obtaining a diagnosis** | 20 days | 10 days |

Which Pathway would you choose? (Tick one box only)

Pathway A Pathway B

# Scenario 7

|  | Pathway A | Pathway B |
| --- | --- | --- |
| **Reservations about seeing a mental health practitioner** | I have major reservations about the child/young person seeing a mental health practitioner. | **I have some reservations about the child/young person seeing a mental health practitioner.** |
| Time to diagnosis | **3 months since the initial contact** | **12 months since the initial contact** |
| **NHS costs of diagnosis** | £10,000 | £5,000 |
| **Days missed from school while obtaining a diagnosis** | 50 days | 20 days |

Which Pathway would you choose? (Tick one box only)

Pathway A Pathway B

# Scenario 8

|  | Pathway A | Pathway B |
| --- | --- | --- |
| **Reservations about seeing a mental health practitioner** | **I have major reservations about the child/young person seeing a mental health practitioner.** | I have some reservations about the child/young person seeing a mental health practitioner. |
| Time to diagnosis | **3 months since the initial contact** | **24 months since the initial contact** |
| **NHS costs of diagnosis** | £1,000 | £10,000 |
| **Days missed from school while obtaining a diagnosis** | 10 days | 50 days |

Which Pathway would you choose? (Tick one box only)

Pathway A Pathway B

Please tell us how easy or difficult you found this section (Section B – Discrete Choice Survey) to complete.

| □ | Very easy |
| --- | --- |
| □ | Easy |
| □ | Difficult |
| □ | Very difficult |

If you found this section of the questionnaire difficult or very difficult to complete, please tell us why in the box below.

Reason?…………………………………………………………………………………………………………………………………………………………………………………………………………………………………………………………………………………………………………………………………………………………………………………………………………………………………………………………………………………………………… …………………………………………………………………………………………………………………………………………………………………………………………………………………………………………………………………………………………………………………………………………………………………………………………………………………………………………………………………………………………………………………………………………………………………………………………………………………………………………………………………………………………………………………………………………………………………………………………………………………………………………………………………………

# Section C - Ranking

Please rank the different aspects of integration of specialised services for functional symptoms in children and young people below in order of their importance to you with 1 being the aspect you consider most important and 4 being the aspect you consider least important.

| ASPECTS OF SPECIALIZED SERVICES | DEFINITION | RANK IN ORDER OF IMPORTANCE  1=most important  4= least important |
| --- | --- | --- |
| Reservations about seeing a mental health practitioner | Refers to extent of reservations about the child/young person seeing a mental health practitioner (e.g., a psychologist or psychiatrist) and the possibility of them requiring psychological/ psychiatric treatment. These reservations might arise from the fear of stigma and prejudice regarding mental health conditions, and the concerns that the symptoms the child/young person experience are not “real”. |  |
| Time to diagnosis | Refers to the length of time needed from initial contact about the child’s/young person’s symptoms with primary care until a final diagnosis is obtained. This does not include potential diagnoses that are explored across the diagnostic pathway. |  |
| NHS costs of diagnosis | Refers to the costs borne by the NHS to reach a diagnosis for the child/young person, for example arising from the number of clinic visits and diagnostic tests needed to reach a diagnosis |  |
| Days missed from school till the final diagnosis | Refers to the length of time needed from initial contact about the child’s/young person’s symptoms with primary care until a final diagnosis is obtained. This does not include potential diagnoses that are explored across the diagnostic pathway. |  |

# Section D - Information about you

In this section we would like to collect some information about you.

**Question 1**

What is your age?

*Tick only one:*

☐18–24

☐ 25-34

☐ 35-44

☐ 45-54

☐ 55-64

☐ 65-74

☐ 75+

☐Prefer not to say

Question 2

How would you best identify yourself?

*Tick only one:*

◻ Female ◻ Male ◻ Other ◻ Prefer not to say

**Question 3**

Are you a single carer of one or more children or young people?

*Tick only one:*

☐Yes

☐No

☐Prefer not to say

**Question 4**

Which category best describes your ethnic group?

*Tick only one:*

☐White

☐Mixed or multiple ethnic groups

☐Indian

☐Pakistani

☐Bangladeshi

☐Chinese

☐Any other Asian background

☐Black, African, Black Caribbean, Black British

☐Prefer not to say

☐Other ethnic group, *provide detail below:*

Other…………………………………………………………………………………………………………………………………………………………………………………………………………………………………………………………………………………………………………………………………………………………………………………………………………………………………………………………………………………………………………………

**Question 5**

Which geographical region of the UK do you live in?

*Tick only one:*

| East of England  East Midlands  London  North East & Cumbria  Northern Ireland  North West of England  Scotland | South East of England  South West of England  Wales  West Midlands  Yorkshire  Prefer not to say  Other [Please specify below] |
| --- | --- |

…………………………………………………………………………………………………………………………………………………………………………………………………………………………………………………………………………………………………………………………………………………………………………………………………………………………………………………………………………………………………………………

**Question 6**

What is your highest level of educational qualification?

*Tick only one:*

☐No formal qualifications or equivalent

☐O level or GCSE, or equivalent

☐ONC or BTEC, or equivalent

☐A level (‘Higher’ in Scotland) or equivalent

☐Higher-education qualification below degree level or equivalent

☐Degree or higher degree or equivalent

☐Prefer not to say

Comment

Please use this box if you would like to tell us anything else about yourself in relation to any aspect of care for functional symptoms.

Comment…………………………………………………………………………………………………………………………………………………………………………………………………………………………………………………………………………………………………………………………………………………………………………………………………………………………………………………………………………………………………………………………………………………………

Thank you so much, you made it ☺! We hope you enjoyed taking part in this study. Your answers will be very valuable to us. If you have any comments, questions or suggestions about this survey or the CENT study in general, please write them in the box below

Comment…………………………………………………………………………………………………………………………………………………………………………………………………………………………………………………………………………………………………………………………………………………………………………………………………………………………………………………………………………………………………………………………………………………………………………………………………………………………………………………………………………………………………………………………………………………………………………………………………………………………………………………………………………………………………………………………………………………………………………………………………………………………………………………………………………………………………………………………………………………………………………………………………………………………………………………………………………………………………………………………………………………………………………………………………………………………………………………………………………………………………………………………………………………………………………………………………………………………………………………………………………………………………………………………………………………………………………………………………………………………………………………………

If you have any questions about this survey please contact:

Lina Massou: em716@medschl.cam.ac.uk

To find out more about the CENT study or to access the results of the study when they become available, please go to: <https://www.nuffieldtrust.org.uk/project/centralisation-of-specialist-health-care-services-a-mixed-methods-programme>

Thank you for taking part.
